# Tuberculosis Recurrence after Completion Treatment in a European City: Reinfection or Relapse?

**DOI:** 10.1371/journal.pone.0064898

**Published:** 2013-06-11

**Authors:** Juan-Pablo Millet, Evelyn Shaw, Àngels Orcau, Martí Casals, Jose M. Miró, Joan A. Caylà, The Barcelona Tuberculosis Recurrence Working Group

**Affiliations:** 1 Epidemiology Service. Public Health Agency of Barcelona, Barcelona, Spain; 2 CIBER de Epidemiología y Salud Pública (CIBERESP), Barcelona, Spain; 3 Departament de Pediatria, Ginecologia i Medicina Preventiva. Universitat Autònoma de Barcelona, Spain; 4 Departament de Salut Pública, Universitat de Barcelona, Barcelona, Spain; 5 Departament de Ciencies Basiques, Universitat Internacional de Catalunya, Barcelona, Spain; 6 Infectious Diseases Service. Hospital Clinic Universitari – IDIBAPS of Barcelona, Barcelona, Spain; St. Petersburg Pasteur Institute, Russian Federation

## Abstract

**Background:**

Tuberculosis (TB) recurrence can be due to reinfection or relapse. The contribution of each to TB incidence and the factors associated with recurrence are not well known. Effectiveness of TB control programs is assessed in part by recurrence rates. The aim of this study was to establish the recurrence rate of TB in Barcelona, the associated risk factors and the role of reinfection.

**Methods:**

A population-based retrospective longitudinal study was performed in Barcelona, Spain. TB patients with positive culture results who completed treatment between Jan 1^st^, 2003 and Dec 31^st^, 2006 were followed-up until December 31st, 2009 by the TB Control Program. The incidence rate of recurrence was calculated per person-year of follow-up (py). Kaplan-Meier and Cox regression methods were used for the survival analysis by calculating the hazard ratio (HR) with 95% confidence intervals (CI).

**Results:**

Of the 1,823 TB cases identified, 971 fulfilled the inclusion criteria and 13 (1.3%) had recurrent TB. The recurrence rate was 341 cases per 100,000 py, 13 times higher than the TB incidence of the general population. Likelihood of TB recurrence at the 1st, 3rd and 5th year of follow-up was 0.1%, 1.4% and 1.6%, respectively. Factors associated with recurrence were HIV infection (HR: 4.7, CI: 1.4–15.7), living in the inner city district (HR: 3.9, CI: 1.3–11.8) and history of TB treatment (HR: 5.2, CI: 1.7–16.2). Genotyping results of recurrent cases were available for 6 patients (3 reinfections and 3 relapses).

**Conclusion:**

The rate of TB recurrence in Barcelona is low and most episodes occur within the first three years. Patients at higher risk of recurrence are co-infected with HIV, living in neighborhoods with high TB incidence or with a history of TB treatment. When available, genotyping results help determine whether the recurrence is due to reinfection or relapse.

## Introduction

Recurrence of tuberculosis (TB) can be due to a regrowth of the same strain of *Mycobacterium tuberculosis* that caused the previous TB episode, known as relapse, or reinfection through a different strain. The data reported suggests that recurrence rate is low in countries with a low TB incidence and mainly caused by relapse of a previously cured TB episode [1]–[3]. The recurrence rate in countries of high TB incidence is elevated and reinfection is the principal cause [4], especially in the presence of high prevalence of coexisting human immunodeficiency virus (HIV) [5]. Studies carried out in countries of medium incidence suggest that relapse more commonly causes recurrence, although the rate of reinfection could still play an important role [6], [7]. Therefore, the relative contribution of recurrent TB on the overall annual TB incidence and the influence of relapse or reinfection is likely to vary depending on epidemiological features of the area [1]–[8].

Information about the epidemiological and microbiological characteristics of recurrent TB is an important issue for public health programs to ensure appropriate health control strategies [9]. Moreover, recurrence rates can be used to assess the effectiveness of TB control programs. Because many large cities in developed countries have recently experienced important demographic changes, related HIV infection and from high-burdened TB countries knowledge of the characteristics and outcomes of TB cases in each population is even more necessary to direct local public health programs. Barcelona had a median TB incidence of 26.3 per 100,000 person-years of follow-up (py) during 2003–2008 but over 100 in some neighborhoods such as the inner-city district, where a significant proportion of residents have low socioeconomic status [10].

The relative contribution of TB reinfection and relapse to the overall incidence and the risk factors associated with recurrent TB are not well-known. This longitudinal study aims to assess the incidence of recurrent TB in a retrospective, large cohort of TB cases and to identify its epidemiological risk factors and microbiological features.

## Methods

### Ethics statement

Demographic and clinical data was obtained from the epidemiological questionnaire used by the Barcelona TB Prevention and Control Program (TBPCP). All data for the study was recorded and analysed anonymously. The data was collected on a routine basis as per the National Tuberculosis Plan approved by the Spanish Ministry of Health and the analysis was carried out retrospectively. Therefore no informed consent was required. Ethics approval was obtained from Clinical Research Ethics Committee of the Institut Municipal d'Assistència Sanitària (IMAS). All data was treated in a strictly confidential manner according to the ethical principles of the Helsinki Declaration of 1964 revised by the World Medical Organization in Edinburgh, 2000 and the Organic Law 15/1999 of Data Protection in Spain.

### Setting

The study was conducted in Barcelona (Catalonia, Spain), an urban area of 100.4 square km, whose census population was 1,508,805 inhabitants in 2008 [11]. The TBPCP has been operating for over 25 years.

### Study design and population

This retrospective population-based cohort study included pulmonary, extrapulmonary, and pulmonary-extrapulmonary TB patients detected by the TBPCP with at least one culture result positive for *M. tuberculosis*, who started treatment between January 1^st^, 2003 and December 31^st^, 2006 who lived in Barcelona during the study period. National and international TB treatment guidelines were followed but patients were not required to complete therapy within 2003–2006. TB cases who completed the entire treatment course according to the recommendations of the European Treatment Outcome Definition were selected and followed to determine the recurrence rate and associated risk factors [12]–[15]. Patients who did not complete therapy were excluded. The follow-up was closed on December 31^st^, 2009. At that date, all cases were classified either as recurrence or censured. The censorship date for each patient was the last day the patient was followed. Censured cases included patients who remained cured, had died, moved away or who were not found (lost to follow-up) at the end of the follow-up period.

### Definitions

A definite TB case was identified using the recommended international definition: a patient was considered to have TB if their culture was positive for *M. tuberculosis* complex. All patients who completed TB treatment, regardless of negative culture conversion, were considered cured., TB recurrence was defined according to the CDC and the Spanish recommendations for TB surveillance [16]–[17] as any new clinical and/or microbiological TB diagnosis in a patient who had completed anti-TB treatment and had been TB disease-free for at least one year since treatment completion [16], [17]. TB disease within 12 consecutive months after the treatment completion was considered the same TB episode. The follow-up time was calculated in reference to the time elapsed since the end of TB treatment until recurrence, death, moved away (transferred), or the end of the study.

### Variables and information sources

All data was obtained from the epidemiological surveys performed by public health nurses on TB cases reported to the Barcelona TBPCP [8]. The Epidemiology Service collects information on all TB and AIDS cases voluntarily notified by physicians and also performs active surveillance for undeclared or subnotified cases coming from microbiology services, hospital discharge reports, city mortality and social service registries. We reviewed the following socio-demographic variables: age, sex, country of birth (Spain or foreign-born), city district of residence (inner-city or other), homelessness, prison history, smoking, alcohol abuse and injecting drug use (IDU). Clinical variables included HIV infection, TB recurrence, and type of TB (pulmonary or/and extrapulmonary forms). Microbiological and treatment variables included smear test results, history of TB treatment, and type and extent of resistance, if any (none, primary or secondary, multi-drug resistance, MDR). MDR was defined as resistant to at least isoniazid and rifampin.

After disease confirmation, study subjects were followed to identify any recurrent TB episode reported to the Barcelona or Catalonia regional programs and/or the date of transfer to another TB Program and to verify their vital status at the end of the study period. Hospital records, primary care records, the city census and mortality registry, and the drug abuse program in Barcelona were reviewed to minimize the number of patients lost to follow-up and avoid duplicate information. At the end of the study, patients were considered lost to follow-up when vital status or data about leaving the city were unavailable.

### Laboratory Methods

*M. tuberculosis* was identified by conventional standardized methods [18] and molecular study of the strains was centralized in one of the six participating centers. Genotyping was performed using the proper standardized protocols for restriction fragment length polymorphism (RFLP)-IS6110 and the IS*6110* fingerprint patterns were analyzed with whole-band analyzer software (version 3.2.2; BioImage, Inc., Ann Arbor, MI) by the unweighted-pair group method with arithmetic means and Dice coefficient. Isolates were grouped into the same RFLP cluster when they showed identical RFLP patterns (equal numbers of IS*6110* bands at identical positions). All isolates with 6 or less IS*6110* bands belonging to an RFLP cluster underwent analysis of a second marker (MIRU 12 or Spoligotyping) [19]–[23].

### Statistical analysis

A descriptive analysis of the cohort was performed with the median and 10–90 percentiles calculated for quantitative variables. Frequency tables and Pearson's chi-squared test were used for categorical variables, as well as two-sided Fisher's Exact tests when expected frequencies were less than five. Non-parametric continuous variables were analyzed using the Mann-Whitney U-test.

The incidence of recurrence in cases py of follow-up was calculated for the general population and for relevant subgroups (IDU, HIV, immigrants, MDR-TB patients, inner-city district residence and history of TB treatment). The rate ratio was calculated to compare the recurrence rate and the median incidence rate of general population during the study period. The denominator consisted of the sum of the follow-up periods from the date of TB completion until recurrence, death, transferred, the last day of follow-up, or the end of the study.

Recurrence curves were estimated using the Kaplan–Meier method. Subgroups of interest were compared using the log rank test, univariate and multivariate analyses were performed using Cox's proportional hazards model and the variables which showed an association (p-value <0.10) or of epidemiological interest at the univariate level were included in the multivariate analysis. *Hazard Ratios* (HR) were used as the measure of association with 95% confidence intervals (95%CI) were calculated and a stepwise forward inclusion approach was used. The proportionality of risks in the Cox model was verified using a Shoenfeld residuals plot and test results were considered to be statistically significant when the resulting p-value was <0.05. All the analyses were performed using SPSS 18.0 and the statistical package R (The R foundation for Statistical computing), version 2.9.0.

## Results

### Cohort selection

**Figure 1:**
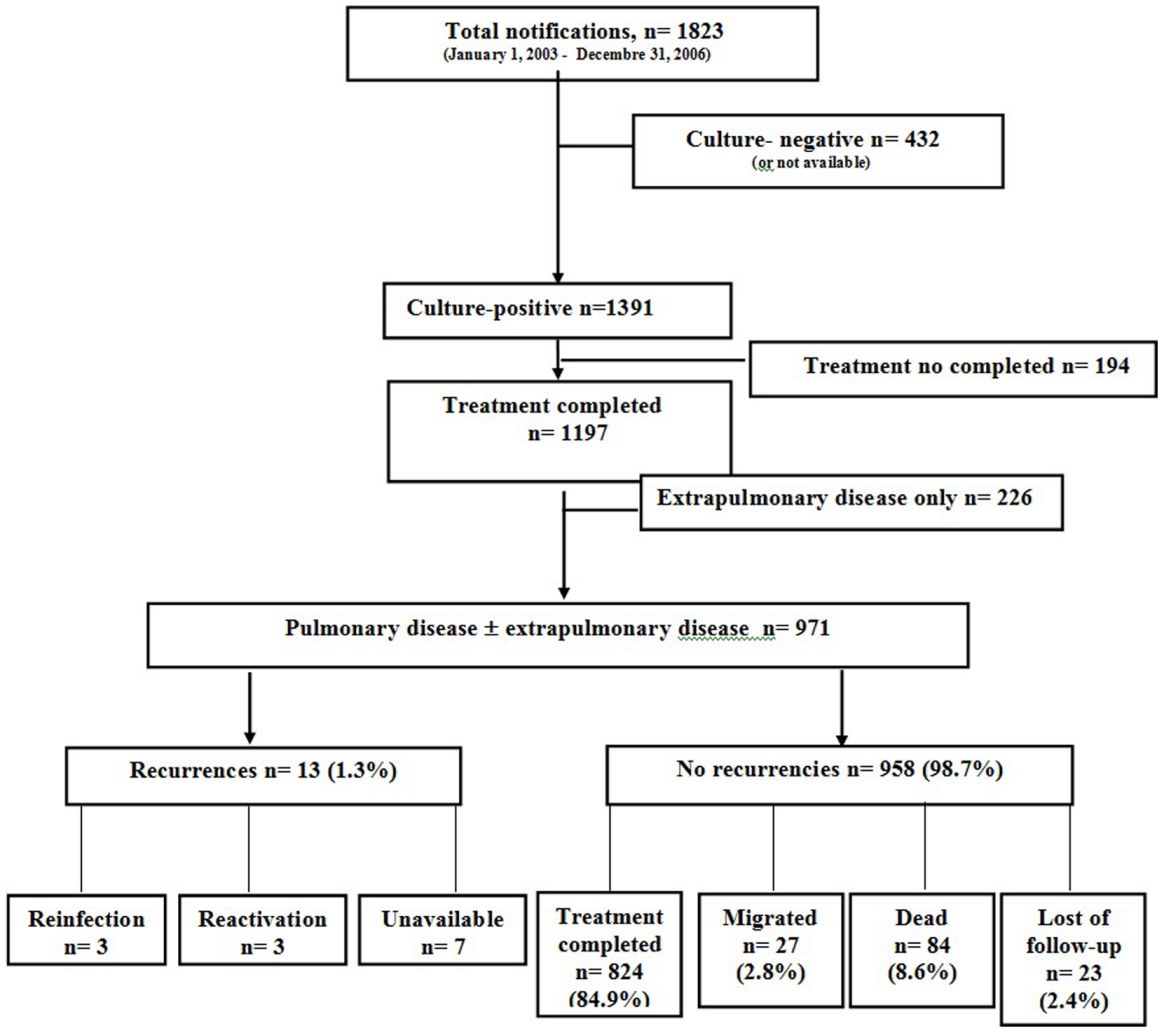
The flow chart of tuberculosis patient selection and evolution. **Barcelona 2003–2009.**

Figure 1 shows the cohort selection flow-chart. A total of 1,823 TB cases were identified during the study period, of which 971 fulfilled the inclusion criteria and constituted the study cohort.

### Cohort description

Table 1 describes the characteristics of the cohort. The median age in the cohort was 38 (Range: 22–70) years and 65% of the subjects were men. Information on drug susceptibility testing (DST) was not available for 194 (20%) patients. A subanalysis of this group didn't show differences with the rest of the cohort except for immigrant status occurred more frequently among those with available DST results. Nine (1%) isolations of MDR-TB were identified and none had extensively drug-resistant TB (XDR TB: resistant to first and second-line TB drugs). Of the 971 cases, 84 (8.7%) history of TB treatment prior to study inclusion. Of these, 5 (6%) had a new episode (recurrence) of TB during the follow-up period. Also of these 84 cases, 51 (60.7%) correctly completed treatment, 22 (26.2%) did not correctly complete treatment and treatment completion was unknown for 9 (13.1%) cases.

**Table 1:** Baseline clinical and socio-demographic characteristics of the cohort and recurrence of tuberculosis. Barcelona, 2003–2006.

	Cohort N = 971 (%)	Recurrence n = 13 (%)	No recurrence (Censure) n = 958 (%)	p-value*
Age years, median (10–90 percentile)	38 (22–70)	43 (22–67)	37 (19–69)	0.40
Sex Female Male	340 (35) 631 (65)	2 (15) 11 (85)	338 (35) 620 (65)	0.16
Country of birthSpainOutside of Spain	629 (65) 342 (35)	9 (69) 4 (31)	620 (65) 338 (35)	1
Residence in the inner city districtNoYesNo fixed residence	777 (80) 171 (18) 23 (2)	7 (54) 6 (46) –	770 (80) 165 (17) 23 (3)	0.02
Alcohol abuseNoYes	718 (74) 253 (26)	7 (54) 6 (46)	711 (74) 247 (26)	0.09
SmokingNoYes	507 (52) 464 (48)	4 (31) 9 (69)	503 (53)455 (47)	0.16
IDU^1^NoYes	912 (94) 59 (6)	11 (85) 2 (15)	901 (94) 57 (6)	0.18
HIV^2^ infectionNoYes	888 (91) 83 (9)	9 (69) 4 (31)	879 (92) 79 (8)	0.02
MDR TB^3^NoYesDrug susceptibility not available	768 (79) 9 (1) 194 (20)	9 (69) 1 (8) 3 (23)	759 (79) 8 (1) 191 (20)	0.11
Direct Observed TreatmentNoYes	786 (80.9) 185 (19.1)	8 (61.5) 5 (38.5)	778 (81.2) 180 (18.8)	0.07
History of TB treatmentNoYes	887 (91) 84 (9)	8 (62) 5 (38)	879 (92) 79 (8)	<0.01

* Chi-square test (Fisher's exact test when an expected value is less than five). Mann-Whitney test was used for age.

1 IDU: intravenous drug use. ^2^HIV: human immunodeficiency virus. ^3^MDR TB: multi-drug resistant tuberculosis.

The median follow-up time was 4 years (2.2–5.9). At the end of the study period, 824 (84.9%) cases correctly completed treatment, 84 (8.6%) died, 27 (2.8%) moved and 24 (2.4%) were lost to follow-up (Figure 1). TB recurrence occurred in 13 (1.3%) cases, with a median time (rank) of follow-up of 2.2 (1–4.5) years before recurrence.

### Recurrence rates

**Figure 2:**
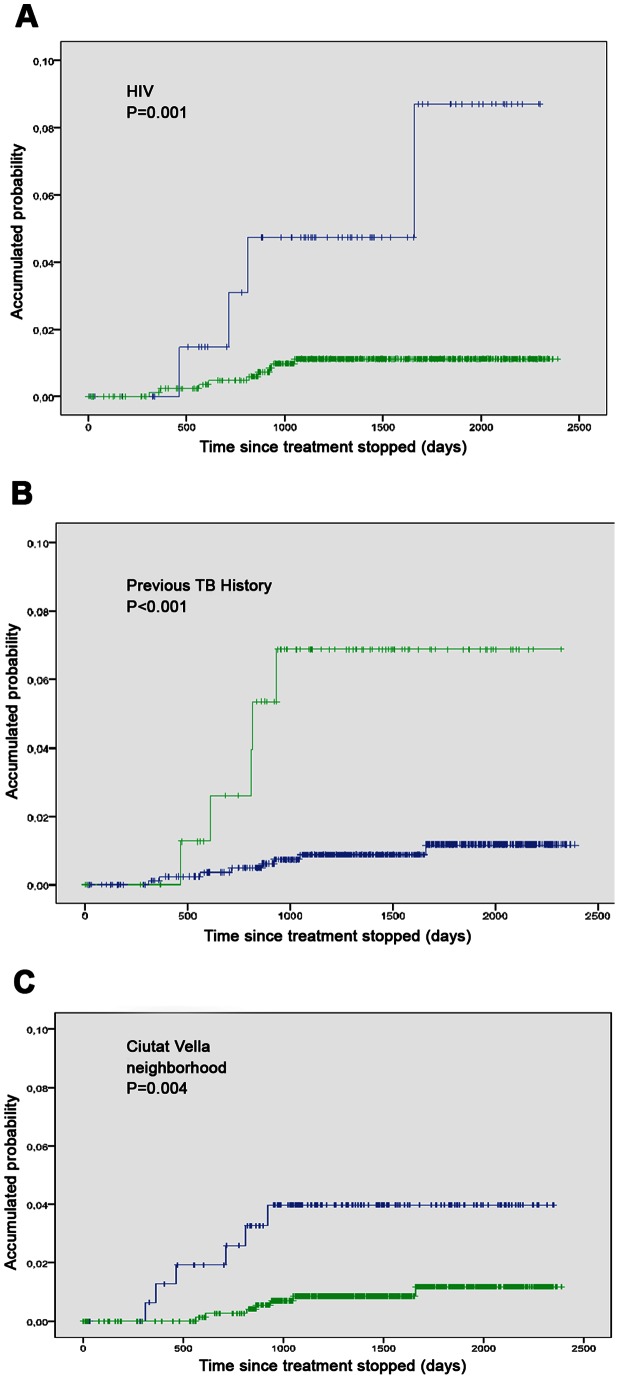
Kaplan-Meier curves of the risk of tuberculosis recurrence among patients with HIV infection, history of TB treatment and who live in the inner-city district. **Barcelona 2003**–**2009.**

The incidence of recurrent TB episodes in the cohort was 341 per 100,000 py. The likelihood of TB recurrence at the 1st, 3rd and 5th year of follow-up was 0.1%, 1.4% and 1.6%, respectively. The incidence rate was higher among patients with HIV coinfection, those living in the inner-city district and in patients with history of TB treatment. The recurrence rate among IDU patients, HIV infected patients and MDR-TB patients were 1,123, 1,492, and 4,166 per 100,000 py, respectively. Recurrence among immigrants was 307 per 100,000 py and among patients with a history of TB treatment was 1,712 per 100,000 py (Table 2 and Figure 2).

**Table 2:** Recurrence rate and rate ratio compared to the median TB incidence in the general population. Barcelona 2003–2009.

	Recurrence cases (n)	Follow-up pŷ	Recurence rate (10^5^ py)	Rate ratio* (CI95%)
Overall	13	3814	341	13.1 (12.1–14.8)
IDU^1^	2	178	1124	43.2 (22.0–64.4)
HIV^2^	4	268	1493	57.4 (43.3–71.5)
Immigrant	4	1302	307	11.8 (8.9–14.7)
Inner-city district	6	614	977	37.6 (31.5–43.7)
MDR-TB^3^	1	24	4167	160.3 (3.2–317.4)
History of TB treatment	5	292	1712	65.9 (53.0–74.8)

* Ratio between recurrence rate and the median incidence rate in the general population during the study period (26×10^5^); ∧py: person-years of follow–up.

1 IDU: intravenous drug use. ^2^HIV: human immunodeficiency virus. ^3^MDR TB: multi-drug resistant tuberculosis.

### Factors associated with recurrence

The following factors were significantly associated with recurrence on a univariate level: living in the inner-city district, alcohol abuse, IDU, HIV coinfection, history of TB treatment, and MDR-TB infection. Living in the inner-city district (HR: 3.9, CI 1.3–11.8, p = 0.02), HIV infection (HR: 4.7; CI 1.4–15.7, p = 0.02) and history of TB treatment (HR: 5.1, CI 1.6–16.2, p<0.01) were identified as independent factors on a univariate level with increased risk for recurrence (Table 3).

**Table 3:** Risk factors for recurrence among of 971 tuberculosis patients. Univariate and multivariate analyses. Barcelona 2003–2009.

	HR unadjusted (95% CI)	p-value	HR adjusted (95% CI)*	p-value
Age≤31 years>31 to 44 years>44 years	0.6 (0.1–2.2)0.8 (0.2–3.1)1	0.400.80	–	
SexFemaleMale	1 3.2(0.7–14)	0.13	–	
Country of birthSpainOutside Spain	1 0.8(0.2–2.7)	0.76	–	
Residence in the inner-city districtNoYes	1 4.3 (1.5–13)	<0.01	1 3.9 (1.3–11.8)	0.02
Alcohol abuseNoYes	1 2.7(0.9–7.9)	0.07	–	
SmokingNoYes	1 2.6(0.8–8.5)	0.10	–	
IDU^1^NoYes	1 3.8(0.8–17)	0.08	–	
HIV^2^ infectionNoYes	1 5.9(1.83–19)	<0.01	1 4.7 (1.4–15.7)	0.01
MDR TB^3^NoUnavailable drug sensitivityYes	1 1.3(0.3–4.8) 13 (1.7–107)	0.68 0.01	–	
History of TB treatmentNoYes	1 7.3(2.3–22)	<0.01	1 5.2 (1.7–16.2)	<0.01

* *Hazard ratio* (HR) adjusted by sex, age and other risk factors. 95% CI: 95% confidence interval. ^1^IDU: intravenous drug user. ^2^HIV: human immunodeficiency virus. ^3^MDR TB: multi-drug resistant tuberculosis.

### Reinfection and Relapse

The molecular study of recurrences was available for 6 of 13 cases. Of these, 3 were reinfections and 3 were relapses. The remaining 7 cases had negative or unavailable TB culture (Figure 1). Relapse was found in two patients with cavitary pulmonary TB who had received six months of anti-TB treatment and one was HIV-positive. The other relapse occurred in a patient undergoing immunosupressive treatment and received eight months of anti-TB treatment. Reinfection was found in a non-Spanish patient who lived in the inner-city, a Spanish-born patient with diabetes, and a Spanish patient with history of TB treatment. None of the reinfected cases were HIV-positive nor IDU.

## Discussion

The overall incidence of recurrent TB in the study was 341 per 100,000 py among patients who completed TB therapy and were considered cured during the study period. Patients who lived in the inner-city district, were coinfected with HIV or had a history of TB treatment had a higher risk for recurrence. Most of the recurrences occurred during the first three years of the study.

A systematic review of prospective cohort studies and randomised clinical trials performed in the 1990s by Panjabi et al. [24] estimated a median recurrence rate of 1,780 per 100,00 py (range 1000–4000) in low incidence countries at 12 months post treatment completion, which is more than five times higher than that observed in our study. Crofts et al. [25] recently investigated recurrences in England and Wales from 1998–2005 and found a recurrence incidence of 660 per 100,000 py among culture-confirmed pulmonary TB cases who completed treatment; TB incidence in the general population was 13 cases per 100,000 py in 2007. Likewise, Dobler et al. [26] reported an incidence of recurrence of 71 per 100,000 py among culture positive patients who had completed treatment in New South Wales, Australia between 1994–2006 (TB incidence in the general population was 6.5 per 100,000 py in 2005). Because the median incidence of TB in Barcelona during the study period of 26.3 per 100,000 py is two to four times the incidence in the studies mentioned above, we conclude that Barcelona currently has a low recurrent TB rate given the overall TB incidence.

We also found a recurrence rate 13 times higher than the TB incidence of the general population, suggesting that TB is more frequent in persons who have had a history of TB treatment. This has already been described in other studies [8], [25], [26]. Our findings also revealed that recurrences do not occur homogeneously among the population. For example, the rate of recurrence among the HIV-infected population was 50 times higher than the incidence of the general population. Physicians should be aware that a history of TB treatment increases the risk of recurrence and that the risk also varies according to the patient profile.

Of the factors determined to be predictive of recurrence, HIV-infection has been previously identified in countries of high and low TB incidence, such as South Africa, China, Spain, Australia, USA, England and Wales [3], [5], [24]–[33]. Among the HIV-infected patients, most recurrences after successful TB treatment are due to endogenous reactivation, probably because of exposure to another strain in low incidence areas is less likely than relapse [18], [31]. As commented by Pettit et al. [18], the higher rate of reinfection among HIV-infected patients may be related to increase in exposure in high incidence areas and subsequent increased risk for disease progression. In our study, only one strain was identified and the rest were not available for the other HIV-infected cases. The rate of relapse found in our study was consistent with that reported in areas with low TB incidence [1], [3], [32]–[34].

Microbiological data was available for three of the six cases of recurrence that occurred in the inner-city district: two were relapses and one was reinfection. The higher risk for persons who live in the inner-city district, where the TB incidence rate is higher than 100 cases per 100,000 py, could be explained by increased contact between individuals due to overcrowding and poor living conditions. Because of this greater incidence observed in the inner-city, we would expect exogenous reinfection to be the principal cause of recurrence, as described in previous studies, [4], [5], [27].

We also found that individuals who have experienced one or more previous TB episode have an increased risk of recurrence, even after treatment completion and cure as of inclusion for the present study. This risk factor was previously identified in a study conducted in South Africa [28], but in association with patients who had defaulted therapy, not who completed treatment. Moreover, the study conducted by Sonnenber et al. [5] among HIV-negative patients showed that the risk of TB recurrence was higher in patients with a history of TB treatment compared to no history of TB treatment. HIV infection was associated with a fivefold higher risk of recurrence in our study, suggesting decreased immunity against TB among this subgroup.

A previous study performed in Barcelona found that IDU cases, immigrants and males were independently related to TB recurrence [8]. These factors were not found to be associated with recurrence in the present study, probably due to the incorporation of directly observed therapy (DOT) to the methadone maintenance program and the role of community health workers for follow-up and contact tracing of immigrants [35]. The lower recurrence rate in this study of 341 cases per 100,000 py compared to 530 cases per 100,000 py among the previous 1995–1997 cohort of could also be due to the extension and free access to highly active antiretroviral therapy (HAART) in Spain since 1996 [8].

Few studies performed in medium incidence countries analyze such a large number of TB cases with a completed therapy in a population-based epidemiological study. However, our study has some weaknesses. First, the number of recurrent TB cases in the study is low and the number of cases without genotyping results is high due to the negative cultures or missing isolates. Second, 23 persons, 2% of the cohort, were lost to follow-up. Most of the lost cases belonged to subgroups with higher risk for recurrence, thus our recurrence rate might be underestimated. However, given the relatively small size of the missing group, it should not have a significant influence on the risk factors found to be associated with recurrence in our study. The recurrence rate could also be underestimated if recurrence occurred outside of Catalonia among patients who migrated during the study period, although analyses do not reveal any differences in characteristics compared to the general cohort. Also, data regarding completion of treatment for a “history of TB treatment” could not be available for some patients. Finally, the lack of information about reinfection from the same strain could cause some cases of reinfection to be deemed relapses. As seen in other epidemiological studies, CD4 cell count and HAART were not reported and could act as confounders among HIV-infected patients and comparisons between our cohort and the general population were not adjusted by age and sex.

In conclusion, our study shows that TB recurrence in Barcelona is low and patients with higher risk of recurrence are those with HIV infection, who reside in the inner-city district, or had a history of TB. This is the first study to our knowledge which found a history of TB treatment as a risk factor for a new TB episode independent of where the patient lives or an immunocompromised status. Further studies focused on this subgroup are required and physicians should be aware that a patient with a history of TB treatment is at higher risk for recurrence, even when therapy has been completed correctly and patient is considered cured.
